# Transactions of Societies

**Published:** 1819-10-01

**Authors:** 


### VIII.

### TRANSACTIONS OF SOCIETIES.

A.] *Cases and Dissections illustrative of Disease of the Brain.* By Samuel Black, M. D. of Newry.

[ Transactions of the Irish Association, Vol. II. ]

Although it may be true, to a certain extent, as Dr. Black justly observes, that the scalpel of the anatomist cannot always discriminate those diversities of organic structure in the brains of the " Lunatic, the Lover, and the Poet," from those of the ordinary race of mortals ; yet, it is evident, that by the *healthy* exercise of the functions of this organ are formed the Poet, the Sage, and the Hero—by it gifted mortals are conducted in the paths of fame, of science, and glory. When these same functions become disordered, unhappy man loses his pre-eminence in the scale of the creation, and sinks to that level which renders the extinction of his being " a consummation devoutly to be wished !"

Our author considers it a principle admirably good in itself, and, in its application, promotive of the interests of science and humanity, that every professional man should contribute his quota towards a general fund, from which we may expect to derive accurate histories of disease, and faithful reports of their appearances after death. And surely, if there be any organ of the body, whose structure, in health and in disease, is more entitled to study and investigation than another, the brain is that organ.

We shall now proceed to the cases themselves, interweaving our own remarks as we go along.

*Case* 1. A gentleman, about twenty-eight years of age, was first seen on the 24th of December 1814, when he complained of pain in his back, but he did not adopt any remedies for it. In the summer of 1815, he bathed three times in the sea, when the pain of the back went off, and was succeeded by severe head-aches. He now came under medical discipline, was prohibited bathing, and prescribed saline laxatives daily, low living, and quietude. He at this time laboured under some mental anxiety. As the season changed, the head-aches increased, and towards the close of the year, there appeared incipient indications of mental derangement. A diffused tumour was now observed on the os frontis, over the right orbit, which was not exquisitely painful on pressure: it was considered to be a thickening of the pericranium.

Early in December he attempted a Journey, but was brought back, and the same night Dr. Black was called to him, and found him stretched on the carpet in violent convulsions. These were succeeded by stupor; on recovering from which, mental alienation was but too evident, for several days. On emerging from this state, he complained bitterly of head-ache. Great depletion, both vascular and intestinal—frontal tumour frequently bled by leeches, and a purulent discharge kept up from its surface. The tumour disappeared, but the head-aches continued, and in three weeks he had a second convulsion. The spring and summer of 1816 were passed in great suffering, and in the month of October the frontal tumour again appeared, and once more receded by the means formerly used.

Early in April 1817, he again consulted Dr. Black, on account of the head-ache, gastric derangement, intestinal torpor, languor, sleeplessness, and emaciation. In May he became completely deranged. On Friday the 26th of June, he was attacked by severe convulsions, succeeded by stupor and insensibility, which continued to recur, at short intervals, till Saturday the 4th of July, when he expired.

*Dissection.* The pericranium, at the site of the tumour, was of a dull, livid, brownish hue, but not much thickened—it was strongly adherent to the skull. The latter adhered very strongly to the dura mater, particularly at that part immediately subjacent to the diseased membrane. On detaching it by the scalpel, about half an ounce of pus escaped, occasioned by a rupture of the dura mater. The dura mater itself, to the extent of a six shilling piece, was five times its natural thickness. On its inner surface were three distinct abscesses, surrounding which was a layer of coagulable lymph, as thick as a wafer. The suppuration did not extend into the brain; but this organ, to the size perhaps of an orange, immediately beneath the diseased membrane, had become a soft and pulpy mass, with scarce a trace of organic structure. There was effusion of water into the ventricles, to the extent of four ounces.

The disease, in this case, was evidently chronic inflammation, and probably a translation of disease from the theca vertebralis, after the sea bathing. The division of the scalp in the first instance might probably have been serviceable, and even had the trephine been applied over the diseased membrane, at a late period, the result might have been more fortunate.

*Case* 2. Miss E. B. ætat. 6, June 10th, 1807, about five days ago, began to complain of her head, with much drowsiness, referring the seat of pain chiefly to the left parietal bone. She had been suspected of worms, and her mother administered some purging powders, which acted well. She had received a blow on the head, three weeks previously, by the falling of a piece of bacon, but the circumstance was kept concealed. Within the last three days the child had vomited frequently. Pupils are dilated in the shade; pulse 94, but very irregular. The progress of the case was marked by heat of skin; intense head-ache; vomiting; stridor dentium; delirium and convulsions. She died on the 19th day of the month.

*Dissection.* Neither the teguments nor pericranium exhibited any diseased appearance, at that part where the injury was inflicted. The vessels of the brain were turgid, and there was an effusion of about two ounces of bloody serum in the ventricles.

*Case* 3. Master C. G. aged nine years, March 11, 1811, had travelled fourteen miles, in a cold day, about a week previously. When he got home, he complained of severe head-ache, and fell asleep. Next day he was drowsy, and complained of much head-ache; vomiting came on, with quick, strong, full pulse; hot skin; white tongue; tenderness in the right hypochondrium on pressure; some difficulty of swallowing; bowels constipated. He now lies in a profound sleep; when roused, he did not appear to notice any thing, but said that the candle hurt his eyes. Pills of calomel and colocynth till the bowels are well cleared.

12th. A drachm of extr. colocynth. comp. fifteen grains of calomel, and a considerable quantity of infus. sennæ produced no effect. An enema brought away one black, costive, and intolerably fœtid stool. Frequent stridor dentium; stupor very great; complains of the candle-light. The purging pills, with James's powder, and infusion of senna were persisted in, and a blister was applied to the head.

13th. Pulse strong and full. After taking ten of the pills, he had two enormously large, black, and fœtid stools. When the bowels began to act, and the blister to rise, the stupor abated, and at present is considerably subsided. The purgatives were continued, and the boy recovered perfectly.

There can be little doubt that the primary morbid impression was here made on the abdominal viscera, and that the cerebral symptoms were consecutive. The state of the brain thus sympathetically induced, might, however, if neglected, have led to all the evil consequences of idiopathic disease in that organ. Dr. Black here states, "his firm conviction that many cases of hydrocephalus are induced by a morbid condition of the chylo-poietic viscera, and still more especially of the liver." P. 292.

We shall conclude our account of Dr. Black's cases, by the relation of one which is calculated to excite painful feelings in the mind.

A fine healthy, lively boy, nine years of age, left his father's house on the evening of Easter Sunday, in company with some servants, who were repairing to a place of vulgar dissipation and amusement. Here some whiskey was given to the boy; but the quantity was not known. He soon got into such a state of intoxication, that it was necessary to carry him home. He vomited on the way, and was put to bed. Dr. B. was called to him at five o'clock next morning, when he found him in a high fever, with flushed countenance; pulse 138, strong and full; the heart acting most violently. He was stupid, insensible, and apparently incapable of speaking. He was taken into a large, cool, and airy room; the head kept erect; the limbs wrapped in flannel; the head, neck, and chest kept sponged with cold vinegar and water, till the animal heat was reduced towards the natural standard. In an hour, the subsultus tendinum and violent starting, which had threatened general convulsions, ceased, the action of the heart was moderated, and the pulse sunk to 108. Purgative enemata were administered, with some effect, and about noon he attempted to articulate, the tendency of which was to complain of his head; twelve leeches to the forehead and temples—bled well. In the evening he drank much whey and lemonade; he had several slight convulsive attacks in the night. Next morning the stupor was considerable; the pulse irregular; the pupils dilated. More leeches and a blister, with calomel and senna, but all in vain. He expired in the evening, fifty-one hours from swallowing the whiskey. No dissection could be obtained.

There is no doubt but pressure on the brain was the cause of death—whether that pressure arose from effusion, or simple turgescence of the vessels.

Dr. Black subjoins to his paper the dissection of four cases of typhus fever. In all these the brain was inflamed or gorged; but in one, there was inflammation also of the mucous membrane of the stomach. Although Dr. Black pretends not to draw general conclusions from these four cases in fever, yet he thinks himself warrantable in protesting *generally* against the administration of wine and spirits, and in recommending early depletion and an antiphlogistic regimen, as best calculated to obviate the occurrences which the scalpel detects in our post mortem researches in fever. In these sentiments, we believe, all unbiassed and enlightened practitioners now coincide.

In this place we may notice, with propriety, a case of inflammation and abscess of the brain, attended with disease of the ear, related by Dr. John O'Brien, of Dublin, in the same work.

A young girl, fourteen years of age, was admitted into the Meath Street Institution, in June 1812, where she was found, by Dr. O'Brien, in a violent paroxysm of delirium; the face red; the eyes wild and suffused, with a contracted iris and small pupil; pulse 120, hard, and irregular. On inquiry, it was found that she had been long subject to a discharge from the left ear, attended sometimes with head-ache; and that the discharge had stopped during the last three weeks, while the head-ache became more violent. Venesection to fourteen ounces; head to be shaved and sponged with cold water; a large blister to the nape of the neck; the ear to be fomented with warm milk and water, and then covered with an emollient poultice; strong purgatives; temporal arteriotomy to six ounces.

2d day. The symptoms somewhat mitigated, but still high. Some convulsive fits in the night. Venesection again.

3d. day. Quite comatose; insensible; pupils dilated; left eye distorted; convulsions; pulse innumerable.

The patient died on the following day.

*Dissection.* Skin and integuments behind the affected ear of a black, or rather dark green colour, extending from the base of the skull half way down the neck. Dura mater strongly adhering to the skull. Pia mater inflamed; its vessels finely injected; ventricles contained very little fluid; but the choroid plexus and lining membrane were highly vascular and injected with blood. Substance of the brain softer than natural. A large abscess was found immediately over the petrous portion of the temporal bone, extending back into the cerebellum, containing matter of a green colour, and very fœtid. The dura mater was destroyed at the bottom of the abscess; the bone itself carious, so that the abscess communicated with the external muscles.

Dr. O'Brien conjectures that the internal membrane of the ear was originally the seat of the inflammation, ending in destruction of the bone. A check to the discharge from cold might easily propagate inflammation to the neighbouring part of the brain, and induce the violent phrenitis just described.

Nothing indeed is more common than the translation of irritations and inflammations to the membranes of the encephalon, by the imprudent application of repellents to eruptions on the head or face. So the sudden suppression of habitual discharges, whether by accident or design, will often determine phrenitis, as every extensive observer must have witnessed.

The remedies suited to cases, such as that related in this paper, are obviously those which produce derivation from the affected part, as topical bleeding; blisters, the warm bath, and purgatives. Opium also, independent of its anodyne qualities, has a remarkable power in lessening mucous discharges.

---

C.] *A Case of Inflammation of the Ear, attended with Symptoms of Compression of the Brain.*

By Richard Grattan, M. D. &c. &c. &c.

[ Transactions of the Irish Association, Vol. II. ]

This is so very important a paper, though ushered in under so humble a title, that we deem it but justice to our readers and to the author, to make it the subject of a pretty long article.

Dr. Grattan observes, that inflammation of the ear is a complaint of very frequent occurrence, and is generally considered of little importance. It is, however, a most painful disease, and we have seen it prove fatal in two instances. We doubt not but most practitioners have, in the course of their experience, met with similar instances.

*Case.* A young lady, who had been subject to inflammatory affections of the throat and face, walked home from a crowded theatre, with her head lightly covered, and in a day or two afterwards was seized with severe pain in the ear, on which a small excrescence had, for some time, existed; cotton and camphorated oil to the ear. At this period, Dr. Grattan examined the lady's head, and found some little fulness round the ear, with a slight purulent discharge therefrom. Fomentations; a purgative, and, if the pain increased, leeches to be applied. Two days afterwards, Dr. G. was called to her, she being reported very ill. The pain had increased, and leeches had given no relief. The pain was now excessive, shooting in different directions from her ear to her forehead. As the Doctor had no doubt but that active inflammation existed, he immediately opened the temporal artery of the side affected, and took away about eight ounces of blood. Five grains of calomel to be taken; succeeded by a castor oil draught, with tinct. assafœtidæ. Shortly after this, she became quite delirious, and soon after stupid. She could, however, point to the head, as the seat of the disease. Head to be shaved, and a sharp blister to the occiput and nape. Temporal artery of the opposite side opened, but only four ounces of blood could be procured. Next day she was worse in every respect. The stupor had increased; her eyes were half open; the pupils dilated, and insensible to light; inability to swallow; moaning; countenance pale; arms inflexibly clasped over the chest. "She had, in fact, every appearance of effusion having actually taken place in the brain." The jugular vein was opened, and ten ounces of blood were drawn off, presenting no buff. Five grains of calomel, and five of James's powder every three hours; a turpentine glyster. In the evening the pulse was fuller, the heat of the skin greater; countenance improved; and she was more sensible. The remedies to be continued. Next day she was quite sensible, but had no recollection of any thing that had passed. Her mouth was now affected with the mercury; an abscess formed behind the ear; the bone was found carious; but she gradually, though slowly, recovered.

Dr. Grattan here favours the profession with some interesting observations respecting the utility of mercury and digitalis, as auxiliary remedies in conjunction with blood-letting, in inflammatory diseases. His opinions are grounded on personal experience of the effects of these remedies, used more freely than is generally practised, and therefore his inferences are entitled to the serious consideration of his brethren.

Dr. Grattan conceives, and justly we think, that there exists a certain tendency of the animal economy to overcome the diseased actions, and restore the affected organ to the free exercise of its healthy functions. This is the *vis medicatrix naturæ*, which Sir Charles Morgan, in his late brilliant attempt to persuade us out of our *minds*, has laughed at, with equal judgment and accuracy of observation. The object of the physician, therefore, says our author, should be to remove, by judicious measures, those causes which tend to maintain the disease in opposition to the exertions of Nature.

Inflammation, Dr. G thinks, is seated in the capillary system of vessels, forming the medium of connection between the termination of the arteries, and the commencement of the veins; vessels which are neither arterial nor venous, but which form a separate system; exercise functions peculiar to themselves; and effect, by their agency on the blood, those various changes, termed secretions. In inflammation, our author thinks there is always congestion in the capillary system, which may be traced to a disproportioned action between the capillaries and the minute arteries by which they are supplied. He thinks, that it is not absolutely necessary that the capillaries should be in a state of debility, but only that their action shall be less than that of the power by which the blood is impelled into them.

When congestion of the capillaries is accompanied by an increased action of the minute arteries, Dr. G. thinks, that acute inflammation is the result; but where this congestion is unaccompanied by obvious increased arterial action, the disease assumes the *chronic* form which, if not relieved, pursues its course, exerting, in appearance, less disturbance, but proving in the end, not less fatal than the former. Thus it is only when, from some local cause, the capillaries lose their power of transmitting the blood to the veins as fast as they receive it from the arteries, or in other words, of equalizing the circulation, that inflammation is produced.

Resolution takes place when the congestion is removed, and the balance of the circulation restored, without any perceptible change in the structure of the part. Effusion, results from the inability of the capillaries to deplete themselves by the usual channels, in consequence of which they pour out a portion of their contents, either on the surface, or into the substance of the part. In suppuration the capillaries appear to assume new functions, and secrete matter more or less purulent.

In respect to therapeutics, Dr. Grattan observes, that when the arterial action is excessive, *general blood letting* becomes indispensible; yet still the influence which it exerts over the capillaries is at best remote, "*and can be considered only in the light of a palliative.*" He thinks it merely moderates the violence of the disease, and affords time to the capillaries to complete the cure. But, as it is in the capillaries that inflammation originates, so whatever remedy acts more immediately *on them,* produces the most certain and decided advantages. For this reason it is that *topical* blood-letting is much more effectual in subduing inflammation, when the situation of the part will admit of it, than *general* bleeding, and much less debilitating to the system. It must, therefore, be very desirable to possess other agents to which we can have recourse, either in conjunction with blood-letting, or subsidiary to it, when its further employment would be no longer admissible. In the diseases of Dublin, at least, Dr. Grattan has found inflammations more successfully managed by blood-letting in moderation, and assisting it by the use of other remedies. After the detraction of blood he has seen digitalis produce the most beneficial effects. To do this, however, it must be given in doses sufficiently large, and repeated at short intervals. Dr. G. administers it in doses of about ten drops, combined with a diaphoretic, every third hour. This plan should be persevered in for two or three successive days. In our author's hands, its administration has been wholly unattended with any danger. Independently of the power which this medicine possesses over the heart and arteries, it has the further effect of increasing absorption from the various textures which are supplied with blood vessels and capillaries. Absorption diminishes the contents of the capillaries, and consequently relieves their turgescence.

"Mercury, like digitalis, acts powerfully on the absorbent system. Although it has a strong tendency to equalize the circulation, and to remove congestion, yet it does not diminish the action of the heart or arteries. Its influence is principally exerted on the capillary system, and particularly on that of the absorbents, which it stimulates more effectually and permanently than any other medicine." P. 340.

Dr. Grattan conceives, and we think him right, that mercury is peculiarly applicable to that variety of inflammation in which the capillaries are turgid from debility; a species of turgescence which is unattended with any very increased action of the heart and arteries, and in which the general system does not strongly sympathize. Here mercury offers important assistance.

In weak and debilitated constitutions, inflammation of the mucous membrane of the lungs, attended with severe cough, and expectoration tinged with blood, frequently occurs in Ireland, when the atmosphere is cold and damp; and in such instances large bleedings commonly produce a strong tendency to effusion, with quick and laborious breathing; tumid countenance; dark colour of the cheeks and lips. In these cases a cautious exhibition of digitalis, giving mercury in as full doses as the state of the patient, will admit, so as to produce its specific effects on the system, using, of course, the other auxiliaries, as blisters, &c. has a most salutary effect.

"It is a remarkable fact, to the accuracy of which I can pledge myself, that I never lost a patient, under the circumstances which I have described, when the system was fairly under the influence of mercury." P. 341.

It is not necessary to induce ptyalism in these cases, though that will sometimes happen, but only to make the mouth sore; and this being effected, the quantity of calomel is to be gradually lessened, or totally withdrawn, as the symptoms appear to be relieved. These observations, says Dr. Grattan, apply to inflammation affecting every other organ as well as the lungs.

We may just remark, *en passant*, that Dr. Grattan appears to overlook too much the great power of mercury in augmenting the various secretions and excretions. We have long come to the conclusion, from pretty extensive observation, that this quality of mercury is among the very first of its salutary operations on the human fabric, when labouring under congestive or inflammatory conditions.

Dr. Grattan concludes with an observation, which is equally worthy of record and recollection. In diseases, he remarks, which depend on chronic inflammation, general venesection, practised moderately, should never be omitted when the symptoms evince a tendency to pass into the acute form. But it ought to be remembered that, in such cases, when acute symptoms *have* actually supervened, the disease ought not to be considered as identical with that which previously existed. Here the vessels assume a new action; and a new disease, in fact, is engrafted on the old, requiring a modified treatment.

"We should not allow ourselves to believe that any disease can be simple and uniform throughout, and preserve the same character, from its commencement to its termination. Every disease must have its different stages; and these and their symptoms are conditions as distinct as the various objects which present themselves in succession, to the view of the traveller during the course of his journey."

By regarding each disease as a series of morbid actions, and adapting our treatment to each particular link, we effectually defend the science of medicine from the degrading reproach of empiricism.

---

D.] *Coincidence of Worms in the Intestines with Hæmoptysis.*
By NATHANIEL RUMSEY, Esq.

[ Medico-Chirurgical Transactions, Vol. IX ]

THE opinion that discharges of blood from the lungs are sometimes produced by the existence of worms in the intestinal canal, originated from observing the repeated occurrence of the two affections in the same persons.

The first case related by Mr. R. was a young woman, about 20 years of age, who had supposed herself pregnant, from the size of the abdomen, which was large and prominent, not resembling the enlargement from a fluid in its cavity, but from a distended uterus. She was not pregnant, however, and in this state she had several violent attacks of hæmoptysis, accompanied with convulsive insensibility and agonizing pain of her side. The blood thrown up was frothy and of a bright red colour, without any indication of its coming from the stomach. Finding that she occasionally discharged portions of tænia, Mr R. prescribed a dose of the ol. terebinthinæ, which operated on the bowels, bringing away a considerable quantity of the worm. The tumefaction went down; the hæmoptysis returned many times slightly, but at length ceased entirely.

The second case was a young gentleman, about 10 years old, who had been afflicted with an obstinate diarrhœa that resisted every measure which Mr. Rumsey could put in force. During the continuance of it, two lumbrici were passed at different times, without any change in the disorder. One night, the family was alarmed by the boy's spitting up about an ounce of blood from the lungs. He was bled, and had no return of it. Shortly after this attack a third lumbricus was passed, and then he got rapidly well.

The third case, and also the fourth, tend to support the supposed coincidence.

There are innumerable facts on record, and indeed are of every day occurrence, to shew that irritation in the digestive organs will produce sympathetic irritation in various and remote parts of the system. Our third number, containing the review of Dr. Wilson's valuable work on Morbid Sympathies, will illustrate this remark. The connection then between worms in the intestines and hæmorrhage from the lungs is by no means incredible, or unreasonable.

---

E.] *Two Cases of Fatal Constipation of the Bowels.* By W. Stoker, M. D.

[ Transactions of the King's and Queen's College, Vol. II. ]

Such cases as the following aie not of very unfrequent occurrence, and their appearances on dissection would probably be more familiar, were there a greater zeal among medical practitioners for post mortem researches.

The first case was an aged maiden lady, who had been for many years habitually costive, but otherwise healthy till the year 1814, when a fit of constipation resisted for many weeks the efforts of the medical attendant. From that time alvine evacuations were kept up by daily laxatives, till within three weeks of the present report. At the commencement of this period, she had, for a few days, a copious flow of limpid urine, followed by a total suppression. Her chief distress now seemed to arise from the painful and enormous distension of the abdomen, which was elevated like a cone with the umbilicus at its apex. No enlargement of any viscus could be discovered. The tumour seemed to be distended by air, which apparently prevaded the whole abdominal cavity. Easiest posture the erect; stomach rejects food; loss of rest and appetite; pulse 90, and full; face flushed; skin dry; strength of voice and voluntary muscles but little impaired. Attributes her present illness to cold caught that morning after taking physic, and states that the swelling of the abdomen increased remarkably during a chilly fit, produced by taking a bottle of aerated magnesia. Nine ounces of blood were ordered from the arm. Pill of aloes, assafœtida, and calomel internally, assisted by tobacco and turpentine glysters alternately. By these means a temporary mitigation of the symptoms was produced, and a scanty discharge of urine and fæces obtained. A kind of borborigmi was now heard through the bowels every three or four minutes, during which the abdominal pain was much increased. No discharge of flatus. For several days the symptoms became progressively aggravated, resisting all the medicinal and mechanical measures suggested or employed by surgeons Richards and Collis, in consultation with Dr. Stoker. On the 1st of June the abdominal tumefaction had arrived at its fullest extent, being like a drum. She expired at half-past nine in the evening.

*Dissection.* On making an incision through the parietes of the abdomen, a very great quantity of fœtid air escaped, and then the belly resumed its natural appearance. The surfaces of the viscera were covered with fæces effused into the abdominal cavity. In the transverse arch of the colon was discovered a circular hole, capable of receiving the end of the thumb, but without any marks of ulceration on the sides of the opening, or of inflammation in its vicinity. No marks of peritoneal inflammation in any part of the abdomen, from the extravasation of the fæces. The rectum, at six inches above the anus, felt extremely hard for a full hand's breadth when slit open; this thickened state was found extending four inches along the posterior part of the intestine, the inner coat being thrown into deep transverse folds, resembling the valvulæ conniventes in form, but exceeding them in depth and closeness of arrangement.

*Case* 2. A married lady, about sixty years of age, was always of a costive habit, and latterly purgatives procured but scanty stools. She declared that she had been now ten weeks without a passage. She was frequently affected with vomiting, and the stomach at present excessively irritable. She is greatly emaciated, and her limbs anasarcous; pulse quick, small, weak; abdomen distended to an enormous size, painful on pressure, and has a tympanitic feel. On examining the rectum by the finger, an obstruction was found high up in it. A small candle was attempted to be passed, but in vain. She died in a few hours.

On opening the abdomen a large quantity of gas, having a fæcal odour, rushed out, and also some yellowish fluid. The whole of the intestinal canal was greatly distended. The cæcum was so much enlarged, that it would have held a gallon of fluid. On its anterior surface there was a small aperture bounded by a circle of a livid greenish colour. It gave exit to a small quantity of feculent matter. The intestines appeared of a dark red colour, but nothing like inflammation was to be discovered. The uterus was cancerous. It had formed a firm adhesion to the rectum and bladder, both of which were in some degree affected by the cancerous disease. A portion of the rectum was found entirely obliterated by the pressure of the uterus.

### F.] *On Apoplexia Cephalica.* By W. Stoker, M. D.

[ Transactions of the Dublin Association, Vol. II. ]

Without setting himself up as a professed reformer of nosological nomenclature, Dr. Stoker thinks himself justified by the following facts, in proposing that Dr. Cullen's third species of apoplexy should be subdivided, and separately considered, under two distinct heads. The first to be named Apoplexia Cephalitica, and the other Apoplexia Hydrocephalica. To illustrate the propriety of this distinction, Dr. Stoker relates a case which he lately attended, in conjunction with his learned friend Dr. Grattan, an outline of which we shall here present to the reader.

A young gentleman, in the eleventh year of his age, laboured under severe fever for twenty-two days, during the last eight of which, and in despite of general and local blood-letting, together with other active means, acute head-ache, delirium, tossing the hands to the head, strabismus, dilatation of the pupils, and impaired vision supervened. Yet, on the most minute examination of the brain made by Mr Kirby, the day after death, no effusion or disorganization could be detected in it, excepting that when the cranium was first removed, "the encephalon seemed to be in size more than proportioned to the bone that contained it, and to expand itself considerably over the under section of the base of the cranium."

Dr. Stoker, and most other careful observers, have noticed all the symptoms which are detailed by systematic writers as denoting effusion or disorganization in the brain, in fevers, and other affections, but where such effusions and disorganizations were disproved by the rapidity with which they disappeared, on removing those causes which inequalize the distribution of the blood in the system, particularly those congestions in the lungs and liver which, our author thinks, impede the free return of venous blood *from* the head, or cause a greater proportion than natural of arterial blood to flow *towards* that organ.

Dr. Abercrombie, in his late "Researches on the Pathology of the Brain," has justly observed, that after death, with all the symptoms of apoplexy, we shall sometimes find effusion of blood, sometimes a small quantity of serum, and sometimes "not the smallest deviation from the healthy structure."* Now it is in the last of these cases that *mere turgescence* of the vessels must have

* Edinburgh Journal, January, 1819. New series.

compressed the *substance* of the brain to such a degree as to occasion death, anterior to rupture or effusion.

The term *determination* of blood to an organ conveys a very erroneous idea to the mind. The heart can have no power to determine more blood to one organ than to another, after the fluid has left the ventricle, although *mechanical* causes in the vessels or organs may cause, as has long been remarked, a greater quantum to move towards the brain, or prevent an adequate return thence. Yet we cannot but consider Dr. Abercrombie's hypothesis of the turgescence of the *artery* in the foramen caroticum, causing an encroachment on the calibre of the *vein* in the same channel, and thus producing simple apoplexy, as extremely unfeasible. Is it likely that the Divine Architect would have left us liable to apoplexy every time that there happened to be any unusual plethora or turgescence of the arteries? Would we not, if this hypothesis were correct, be affected with apoplexy in every hot stage of fever, when the arterial system is, beyond all comparison, fuller than the venous?

But the fact is, that the great and paramount cause of vascular turgescence in the brain, and in every other organ, must be sought in the nerves, and in the vital power of the vessels themselves. Irregularities of the nervous influence, and, as it has been called, the *tonicity* of the vessels, will occasion an engorgement of the latter in any organ whatever; and, in this respect, we may take a lesson from our medical brethren on the continent, who have applied the term *apoplexy* to congestions in the lungs, liver, or even the expanded tissues themselves. And why not? Have we not, for instance, in the lungs, a turgescent state of the vessels, sometimes fatal, as in asthma; but more frequently relievable? Have we not serous effusion there, as in serous apoplexy? Have we not *sanguineous* effusion, sometimes fatal, sometimes not, as in sanguineous effusion on the brain?

A diagnosis between these varieties, (cephalic and hydrocephalic) as Dr. S. observes, would be a great desideratum in a practical point of view. It would prevent a two-fold evil which has hitherto existed, fallacious hopes being indulged in one form of the disease, from means that removed the other; while their failure in the former deprive them of their merited repute as remedies in the latter.

*Case* 2. Miss C. hitherto an animated child, and now four years of age, became, on the 25th of October, 1817, restless and fretful; nauseated her food, with hot and dry skin, flushed face, costive bowels, and pain in the head. Strong purgatives and leeches had been used, before Dr. Stoker's attendance. On the 3d of November he found the patient constantly moaning and agitating the limbs, the hands being often applied to the head; frequently uttering a shrill scream, especially when moved; face pale, and somewhat jaundiced; skin dry, but not very hot; pulse rather indistinct, and unequal, but about 160; muscles of the abdomen very rigid; preternatural fulness in the right hypochondrium; apparent loss of vision; the direction of the eyes not affected by the candle light; pupils dilated and insensible to light; some strabismus; eye balls have a glazed appearance; stools very scanty, and green; urine scanty; stomach quickly rejects solids or liquids; no sleep the last 24 hours. Warm bath at 98° for ten minutes, during which a sponge of cold water was applied to the head. On removal to bed, the pulse was more distinct, and muscular rigidity less. Turpentine enemata were directed, and as soon as they could be taken, small doses of calomel and scammony to be frequently repeated. The warm bath, and cold to the head to be used again in the evening, and six grains of Dover's Powder and two of James's Powder to be given on being removed from the bath, and a similar dose at midnight. Blister to the right hypochondrium.

November 4th. The symptoms somewhat mitigated, and the same remedies continued, with the addition of a table spoonful of the infusion of green tea every two hours.

5th. Relishes the tea, which seemed to have relieved both the coma and gastric irritability. The pupils are still dilated, and cornea dull. She knows her mother's voice, however, to day. No alteration in the treatment, except the substitution of infusion of roses and Epsom salts for the purging powders.

6th. The calomel and scammony were resumed, and the other remedies to be continued as before.

7th. Bowels well freed; fæces natural; urine free; tongue loaded, but soft; eyes and countenance improved in appearance; pupils contract slowly on the approach of light; pulse 120. From this time convalescence gradually advanced, and the recovery was complete.

Dr. Stoker concludes his paper with some observations on the infusion of green tea in comatose affections, a remedy which he has found very useful, especially where such states are connected with fever, or have succeeded the use of opium. In these cases it has agreed well with the taste and stomach, rousing vital energy without increasing febrile excitement; promoting healthy secretions, especially that of the urinary organs. In illustration of this statement, he relates a case, of which we here present an outline.

A married lady, thirty years of age, who had weaned a healthy child a month previously, was, on the 9th of February, attacked with fever, and on the 20th of that month, the day on which Dr. S. first saw her, she laboured under the following symptoms: anxious countenance; black circumscribed flush on the cheeks; eyes suffused, and their vessels turgid; tongue brown and dry; teeth sordid; low delirium; pulse indistinct; pervigilium; frequent moaning; general debility; hands tremulous; lies on her back, sliding down to the foot of the bed; skin clammy, and covered with large dark petechiæ. Her bowels have been freed by calomel, turpentine, and castor oil, assisted by glysters; alvine discharges very dark and offensive. A table spoonful of yeast with two of camphor mixture every second hour; the head to be shaved and washed with vinegar and water, as also the arms, face, and shoulders. Claret and water for drink, with soda water, &c.

21st. No very remarkable change in the symptoms; face flushed; pulse 140, but firmer; more delirium; temporal and carotid arteries throbbing violently. Five ounces of blood to be taken from the temporal artery, and a blister to the nape of the neck. Other remedies as before. 22d. Every symptom worse; pulse 150, and feeble; tongue dark brown. 23d. Had three convulsive fits, and all the symptoms of the very worst order. On the 24th she lay in a comatose state with hiccup and vomiting; suppression of urine. Two table spoonfuls of a strong infusion of green tea to be given every two hours, and a weak infusion of it for common drink. *Evening.* Much more animated; swallows easily; no vomiting; passes urine; pulse 100; bowels free; relishes the tea. From this time she convalesced rapidly, and completely recovered. From these cases it appears that the praises bestowed on the infusion of this celebrated plant by Kien Lien, in his Epic Poem, are not so exaggerated as they have long been considered. Certainly no medicine can ever be so universally agreeable to the patient as this.

G.] *On the Use of the Actual Cautery as a Remedy for the Cure of Diseases.* By J. P. MAUNOIR, Professor of Surgery in the University of Geneva.

[ Medico-Chirurgical Transactions, Vol. IX. ]

NOTWITSTANDING that the application of fire to the human body was considered by the ancients, as it is still so by our continental brethren, as a powerful remedy in numerous diseases, yet, in England, such is the prejudice against the actual cautery, on the part both of patient and practitioner, that it is very doubtful if it ever come into general use, even if its powers be acknowledged. Yet, as we are very frequently baffled in our remedial measures, and as we may and do, occasionally meet with patients whose firmness of nerve will permit them to bear an apparently cruel remedy, rather than undergo continual sufferings; and as, in hospital practice, the cautery may be easily introduced [indeed, it has lately been practised in a public hospital in this metropolis] so we think that our countrymen should not be entirely led away by the prejudices of Mr. Sharp, whose oracular influence seems to have abolished the actual cautery in these islands. With the view, therefore, of exciting the attention of British surgeons to this point, we shall glance at those cases which Professor Maunoir has brought forward, illustrative of the powerful effects of the cautery, when every other remedy had failed.

*Case* 1. A young Savoyard, tall and well made, had for many years been afflicted with local scrofula. A hard, red string of glands suppurating in seven or eight places, extended along the lower jaw and upper part of the throat from ear to ear. All remedies had been tried in vain. Fatigued by their inutility, M. Maunoir proposed the actual cautery, which was immediately assented to. The fistulæ and ulcerated glands were cauterised with an iron heated to the greatest degree, and the difference in their appearance, after the sloughs had fallen off, was astonishing. Some of the ulcers cicatrized, and others resisted the measure, which was then a second time resorted to, and a perfect cure was the consequence. The enlargement of the glands totally disappeared in six months after the cicatrization of the ulcers.

*Case* 2. About the same time, M. M. attended a villager, in the Canton de Vaux, whose left thigh was burrowed by sinuous ulcers. In consequence of the frequent application of the olive-shaped cautery, not only the fistulæ were cicatrized, but the thigh and leg so far recovered their strength as to be nearly on a par with the other limb.

*Case* 3. Mr. P. an English gentleman, had, for fifteen years, an ulcer on the lower lip, with every character of cancer. He had been under several eminent surgeons in London, without gaining any relief. The ulcer was deeply cauterized, and the operation gave infinitely less pain to the patient than he expected. The process was renewed twelve days afterwards, and the patient was cured in the space of three weeks, of a disease that had resisted fifteen years' treatment.

*Case* 4. Mr. M. D. aged forty-five, of dissipated habits, had been tormented with rheumatism to a violent degree, in the lumbar region. The spine began to bend, and formed an elevation near the sacrum. His legs grew weaker from day to day, and at the last became totally useless. All kinds of internal medicine were used in vain. The cautery was applied to the lumbar region, so as to make about half a dozen sloughs, a few inches long, and half an inch wide. From that time the patient recovered by degrees the movement of his inferior extremities—then was enabled to walk with crutches, and lastly without any help at all.

The most important precaution to observe in employing the cautery is, to use the iron only when it is nearly white with heat, and to apply it instantaneously, so as to destroy the parts it touches, while it scarcely heats those around them.

---

H.] *Two Cases of Aneurism, in which the temporary Ligature was employed.* By B. Travers, Esq. &c.

[ Medico-Chirurgical Transactions, Vol. IX. ]

In the first case, the brachial artery was tied an inch and a half above the bend of the elbow, with a noose ligature, so as to render its removal practicable, if judged expedient. The pulsation in the radial artery immediately ceased. The ligature was removed fifty hours after the operation. The wound had adhered, except round the ligature, which was imbedded in coagulable lymph. He was discharged cured. In the second case, which was popliteal aneurism, the ligature was applied in the usual place, and removed twenty-seven hours after the operation. No pulsation could be felt in the sac. This operation failed, and another ligature was obliged to be put on the artery above the former, and allowed to come away spontaneously, and the man was cured.

The first case, Mr. Travers observes, shews that the residence of the ligature upon the artery for a period of fifty hours, as completely answers the purpose of its application as though allowed to remain until thrown off by the natural process.

In the second case, the suspension of the pain, and the diminished strength of the pulsation for a month following the application of the temporary ligature, only shew that an impediment to the current of blood in the artery had been effected; but the hope of cure was fallacious, and no such expectation, thinks our author, ought ever to be entertained while the pulsation, in any degree, of the aneurismal sac continues. The establishment of a collateral circulation is insufficient for the cure of the aneurism, while the sac continues to receive a full jet of blood. Mr. Travers has seen a case in which the artery being obliterated below the sac, collateral circulation was established; yet the pulsation of the sac was undiminished, and the progress of the disease unretarded. On the other hand, the arrestation of the pulse in the sac, unless attended by a diminution of its volume, and freedom from pain, affords no just ground for supposing that the natural cure of the aneurism is in progress.

Mr. Travers has determined to relinquish the use of the temporary ligature, of which, he confesses, he had formed too favourable an opinion.

---

### I.] *Successful Operation of Paracentesis of the Thorax.* By Nicholas Archer, M. D.

[ Transactions of the Irish Association, Vol. II. ]

The successful cases of this kind are so thinly scattered in the Records of Surgery, that the following instance will be read with much interest.

The subject of the operation was a gentleman, aged 41, who, till within these last three years, had enjoyed good health, and was strong in habit and temperate in living. About three years previously to our author's seeing him, he had an attack of pleuritis, which yielded to bleeding, blistering, and the antiphlogistic regimen. Shortly after this attack, he began to complain of a short, dry cough; a sense of heat in the right side, not amounting to pain; difficult breathing on using any exertion; palpitation and lividity of countenance, except when tranquil. He removed to Portugal, by the advice of his physicians, but returned home after a year's sojourn there, without experiencing any relief. While in Portugal, he thought that he could perceive the motion of a fluid in the right cavity of the thorax, but this idea was scouted by his medical friends.

On Dr. Archer's visiting him, all the above-mentioned symptoms existed in an aggravated degree, with dark livid countenance; pulse at 130; great emaciation, yet his breathing was but little disturbed, except on using exertion. Dr. A. examined the chest minutely, and on pressing the fingers strongly between the ribs of the right side, while the patient gently agitated the thorax, he distinctly felt an undulating motion; and on applying his ear close to the part, he could hear a noise like that produced by shaking a small cask not quite full of water.

Dr. A. proposed paracentesis, to which the patient submitted with the greatest alacrity. The operation was accordingly performed, and eleven pints of an inodorous fluid, resembling whey, were gradually abstracted. The tube of the canula was frequently obstructed by a solid substance; but, on the introduction of a probe, it would pass off. These substances were discovered to be small branches of the bronchi in a shrivelled decayed state. While the fluid was drawing off, his pulse gradually diminished in quickness and increased in fulness. At the close of the operation, they rested at 86. For some days after this, the discharge from the orifice amounted to nearly two pints daily, of the same kind of fluid; but it gradually lessened; his breathing became free; the lividity of his countenance disappeared; his appetite mended; he gained flesh every day; was able, in a few weeks, to walk and ride out in the open air, and his cough nearly subsided.

At his own suggestion, a slightly astringent lotion was thrown into his chest, through the open orifice; at first, consisting of lime water mixed with rennet whey; and, in a short time, they ventured on a weak solution of sulphate of zinc in rose water, with decided advantage. In four months all discharge ceased, he gained strength, and enjoyed tolerably good health for three years.

K.] *Case of an unusual Termination of Psoas Abscess.* By SAMUEL WILMOTH, Esq. Lecturer on Surgery, &c.

[ Transactions of the Irish Association, Vol. II. ]

MARTIN CARROL, aged twenty-two years, a strong athletic young man, was seized, in the beginning of 1814, with a pain in his loins, after lying on a damp bed. He was repeatedly blistered without relief. In a twelvemonth his health began to decline. He was now frequently attacked with chills towards evening, with heat and restlessness after going to bed; perspiration towards morning; bad appetite; great thirst; pain in the back much increased by striking the foot against any thing.

A tumour now appeared in the left groin, which was the cause of his applying to Mr. Wilmot. This tumour was about the size of a tennis ball, but flat, and immediately under Poupart's ligament, accompanied with a swelling above it. The integuments covering the tumour were a little discoloured, and a distinct fluctuation could be felt within it; it was slightly affected by the horizontal posture and coughing. He would not permit an operation; but after the lapse of a year, he returned, when the tumour, though increased in size, had, nevertheless, lost all the characters of an abscess. It was now of a conical shape, measuring nearly fourteen inches at the base, and from the base to the apex seven inches. All sense of fluctuation within the tumour was now gone, and it had a tympanitic, elastic feel. The patient's health was now completely re-established; the hectic symptoms, pain and weakness in the back, had disappeared without medical assistance. But the tumour continued rapidly to increase, producing considerable inconvenience.

On accurate examination, it was found that the contents of the tumour communicated freely with the abdomen, by means of a narrow conduit running in the direction of the psoas muscle; through which the whole fluid of the tumour could be pressed up into the belly, making it evident that the external sac was an elongation of the internal cavity. On removing the pressure, the sac suddenly became distended to its usual size, although the patient lay in the horizontal posture; from which circumstance, together with the elastic and tympanitic feel of the tumour, it was evident that it was now filled with *air*. The plan of cure was either to puncture, and procure adhesion of the parietes of the sac; or, by pressure, procure the absorption of the contained air. A bandage and compress, wet with a strong decoction of oak bark and allum, were applied, and produced a considerable corrugation of the skin. In about three weeks, the air was almost entirely absorbed, and the sac considerably diminished. A truss and pad were fixed on the part, and four weeks afterwards he left the hospital perfectly free from swelling.

---

L.] *An Account of the Extirpation of a Tumour of the Neck, engaging the Parotid Gland.* By RICHARD CARMICHAEL, M. R. I. A. &c.

[ Transactions of the Irish Association, Vol. II. ]

IT is well known to our anatomical readers, that the parotid gland occupies the deep recess between the ramus of the lower jaw, and the auditory and mastoid processes of the temporal bone, covered externally by the cervical fascia, platisma myoides, and skin; while deeply buried in the centre of the gland, the external carotid artery sends off important branches to the jaws, the face, the temple, and the ear. Almost in contiguity with its internal surface are, the internal carotid artery, jugular vein, par vagum, great intercostal, and other nerves.

Any operation for the removal of such an organ, few surgeons will attempt, without anxiety and trepidation, especially when they read the following sentiments.

" No one, surely, (says Mr. Abernethy) would think of cutting out the parotid gland." " The cutting out completely the parotid gland, (says Mr. John Bell) is a thing *quite impossible*, since the greatest of all the arteries, viz. the temporal and maxillary, lie absolutely imbedded in the gland." *Anatomy, vol. ii. p.* 293.— It is true, that, with his usual consistency, Mr. John Bell boasts, after the above declaration, of having *frequently performed* this very operation!!!—Mr. Burns, in his Surgical Anatomy, p. 267, observes, that—" Extirpation (of the parotid gland) is quite out of the question. Its impracticability is proved by reviewing the connexions of the gland. It is an operation which, *in no situation, and under no circumstances*, ought to be undertaken."

But surely some exaggerated sense of danger, some hypothetical phantom, the aerial nature of which, it is the object of these preliminary observations to demonstrate, must have haunted the imaginations of these gentlemen, and crossed and disturbed their intellectual vision while contemplating this subject.

Two principal arguments have been urged against this operation. The first is the intimate connections of the external carotid artery with the gland. But what British surgeon will now dread the consequences of destroying the external carotid, or tremble at hæmorrhage from any vessel accessible to the scalpel and ligature? The second objection is more specious. No one, it is contended, can clear away the substance of the parotid from all the deep recesses to which it penetrates; and imperfect removal of the organ, when invaded by malignant disease, must be useless, since the progress of the poison and reproduction of the morbid action will not thereby be checked. But one fact is worth a dozen of arguments; and fortunately we are now enabled to lay before the reader *two* well authenticated cases, in solution of this important surgical problem.

Our reason for *reverting* to the first of these cases is, that at the period of its promulgation, this Journal, from a combination of circumstances, was in the very infancy of its *circulation*, and consequently its contents but little diffused—a period, by the bye, when some of our *kind* cotemporaries assigned, with equal liberality and *prescience*, "*a short and feverish existence*" to a Journal which, Antæus like, has risen higher by falling—expanded by pressure—increased its velocity by diminishing its pace—and strange to say, has gained in territory by the dismemberment of its constituent parts.

In the 6th No. of such a Journal (June 1816) Dr Shirley Palmer, a respected coadjutor, at that time, has detailed a most interesting operation, in which, we believe, he performed an important part; and where the parotid gland was extirpated, and the great branches of the external carotid divided, and safely, though with difficulty, secured.

Previously to the operation, the tumour had increased in size so considerably, as to seriously impede the motions of the lower jaw. The numbness and lancinating pains were great; the knotty irregularities of the surface were distinctly marked; the integuments of the centre rapidly verging towards ulceration. Indigestion and slight febrile paroxysms indicated the approaching ravages of the local malady on the system. The operation having been finally determined on, shall be detailed in the words of Dr. Palmer himself.

"The morbid portion of integument was, in the first place, insulated by two curved incisions meeting each other above and below, at an acute angle. Upon exposing the organ towards the masseter muscle, the substance of the parotid itself, indubitably recognized by the origin of its excretory duct, was seen knotty and enlarged. Our minds had been duly prepared for this discouraging circumstance; our resolution was irrevocably fixed. The integuments covering the posterior part of the gland were now dissected back. On detaching it towards the mastoid process, the posterior artery of the ear was divided, and bled, for a time, profusely. The temporal, transverse fascial, and internal maxillary, successively sprang on insulating the gland in other directions. The hæmorrhage was great; but pressure on the mouths of the vessels prevented it from becoming excessive. At length the whole of the morbid organ was detached, except at the base. This was the precise period for the application of a ligature; but the hope of speedily terminating a bloody and protracted operation, prevailed with us over every other consideration. The final detachment of the gland was effected by a stroke of the scalpel, and the gush from the external carotid was most appalling. I thrust a sponge into the wound, and retained it with considerable force. The man, whose countenance had long been bleaching, now became faint. The operator, availing himself of this favourable moment, fixed a tenaculum into the artery. I attempted to secure it, but this was rendered exceedingly difficult by the depth and narrowness of the wound—the ligature slipt. Two other attempts were equally abortive. The man was now recovering from his syncope—the artery beginning to pour out a frightful torrent. The situation of the poor fellow was, at this moment, most critical; our own embarrassing. At last, amid such a deluge of blood as I hope that these hands may never again be embued in, the ligature was fortunately fixed." 461.

The man perfectly recovered. We have brought forward this interesting case, confident that Mr. Carmichael knew nothing of its existence, otherwise his acknowledged candour would have taken some notice of the operation. We now proceed to the case which stands at the head of this article.

The patient was a man of temperate habits, about 40 years of age. The tumour was first observed about the size of a kidney bean, lying below the ear, fourteen years previous to the operation. It increased slowly, and in four years had attained the size of an egg, at which time it was removed by an eminent practitioner. The tumour reappeared, and increased with greater rapidity than ever. At the time of Mr. Carmichael's operation, (14th December, 1817) the tumour was five inches, measured vertically from the external ear to the neck, on which it descended — horizontally, immediately below the ear, three inches and a half; and at its inferior part, full five inches. It was of a firm consistence; but not of that cartilaginous hardness which cancer, in this situation, usually possesses.

Our author began by making an incision the entire length of the tumour, along the internal edge of the sterno-cleido-mastoideus muscle. It was then carried considerably below the tumour, for the purpose of exposing the common carotid, in order to admit either of compression or ligature, should such a measure be found necessary. Two threads were passed round the external jugular, and the vein divided between them, to prevent any embarrassing hæmorrhage from *that* vessel. The artery, as well as the mastoid muscle were much displaced by the pressure of the tumour, being pushed nearer the trachea than natural. The integuments were next dissected back, at either side of the incision, so as to expose the external part of the tumour. It was then detached from the meatus auditorius, and ear, and from the mastoid process behind, and from the angle of the jaw before, to which parts it firmly adhered. Mr. Carmichael now used the handle of the scalpel and his fingers to separate the tumour from the deep-seated parts lying between the temporal bone and ascending process of the lower jaw, where it was imbedded to an unexpected depth. A firm band or root, connected with the deepest part of the tumour, having a large artery distinctly beating upon its surface, was found to resist all efforts to disengage it; and it became absolutely necessary to have recourse to the knife. Previous to this division Mr. Todd was prepared to compress the carotid trunk, in case of hæmorrhage. The band was then divided with the buttoned bistoury, close to the tumour. Instantly an alarming gush of blood, evidently from a large artery, followed the division. The danger was the more imminent, as the pressure which Mr. Todd applied, with all the force he could exert, was incapable of repressing the torrent. Mr. Colles plunged a sponge to the bottom of the wound, and firmly pressed on the bleeding vessel, while Mr. Carmichael made a horizontal section of the tumour till he arrived at the cavity filled by the sponge, with the view of exposing as quickly as possible, the mouth of the bleeding vessel. This was accomplished in sufficient time to save the patient's life; and a large artery, which was probably the trunk of the fascial or labial, was tied in two places; that is, at each side of an orifice, resembling the vent of an organ-pipe made by the knife, not by dividing the artery, but by taking off a slice of its surface. When the ligatures were fixed, the pressure from the carotid was removed, and no further hæmorrhage occurred. That part of the tumour which remained was found adherent to the bones of the basis cranii, and to the transverse process of the atlas. Mr. Carmichael's friends dissuaded him from proceeding farther; he had the courage to proceed. With the fingers Mr. C. removed that portion which was fixed to the temporal bone betwixt the mastoid and stiloid process. This was accomplished with great pain to the patient, as the trunk of the portio dura of the seventh pair was separated with the diseased mass. Another portion was removed in a similar manner, which was fixed to the transverse process of the atlas; but another small portion remained so firmly fixed to the bones at the basis of the skull, that it seemed more prudent to pass a ligature around its base than to make any farther attempts at its extirpation. The wound was now cleared of coagula, the edges brought together with adhesive straps, and this strong-minded individual walked without assistance to his bed in an adjoining room. After some alarming symptoms the man ultimately did well.

Mr. Carmichael concludes that the success of this operation (strengthened by that of Dr. Palmer's already narrated) "proves the possibility of extirpating the parotid gland." Should he be called upon, however, to perform so formidable an operation again, he would, in the first instance, pass a ligature under the carotid trunk, which might be tightened or not, as occasion should require.

It is needless to say, after the full account which we have given of this operation, that it reflects the highest credit on the intrepidity, dexterity, and prudence of the very able operator.

---

M.] *Case of successful Cæsarean Operation.* By J. J. Locher, M. D. Town Physician of Zurich. Communicated by Dr. Albers, of Bremen.

[ Med. Chir. Trans. Vol. IX. ]

Dr. Albers considers it a most remarkable circumstance, that in England, where the boldest operations of every description have been performed with success, the Cæsarean operation should never have been attended with the same fortunate result as in other countries. We do not attribute this to any thing in the operation itself, but to the constitution and climate of the patient.

Dr. Locher was summoned to a woman in labour, who had been ricketty in youth; and who, from the os ilii downwards, was entirely crooked. She had spasmodic rather than labour pains. The os uteri was a little open. Behind it, though in an oblique position, and extremely high, the head of the child could be felt. The pelvis was found distorted. The forceps were in vain attempted to be applied. As the child was still alive, it was determined to save the life of *one* at all events, and therefore the Cæsarean operation was agreed to.

The incision was made directly upon the linea alba, and no blood-vessel of any importance was divided. Immediately beneath the navel the skin was pinched up into a fold, both it and the adipose membrane cut through, and the cut continued downwards to the length of from eight to ten inches. The sphere of the uterus now appearing, extended the fat edges of the incision, so that there appeared a considerable vaulted surface of the womb. There protruded also a portion of small intestine. In order to avoid the placenta, a somewhat uneven part of the uterine surface was selected, and there a small incision was made, so that the operator could introduce the index finger of the left hand to serve as a guide for the progress of the knife. The uterus was then cut open from six to eight inches along the finger. Immediately the child presented itself, together with its membranes, yet without any water. The hæmorrhage till then was a mere nothing. The nearest part of the child was an arm: this was first disengaged from the uterus, and then one part after another in succession, and last of all the head. The infant immediately moved, and evinced its life by loud cries. In the right side of the womb was found the placenta, which was removed. At this period, a violent bleeding took place from the bottom of the uterus, and the blood was absorbed by means of a sponge. The external teguments were joined by five sutures, covered with lint and adhesive plaster, and the whole confined with a couple of compresses and a broad bandage. The mother did not faint; on the contrary, joy was depicted in her countenance. She ate some good broth with great appetite, and she was prescribed a draught with laudanum and tincture of cinnamon. During the night she enjoyed quiet sleep at intervals, and only complained of a burning heat in the wound. The blood began to flow from the vagina; she passed water in the natural way. The belly was protuberant and tense, but not very painful to the touch. The second and third days passed well; the lochia flowed in due order; the belly grew softer, but no stool passed, though glysters were exhibited. Decoct. tamarindorum.

In the night between the third and the fourth day, a stool was passed, with much flatulence. The belly was soft; the patient, upon the whole, well, quiet, and without fever. On the 4th, in the morning, every thing appeared flattering; but towards noon, Dr. L. was sent for in haste, as the patient was supposed to be dying.

"On my arrival, I found the woman in a very indifferent situation. She experienced violent convulsive spasms, particularly in the head. She had a staring look; cold extremities; cold sweat on her brow; the urine had been discharged involuntarily. She recollected nobody; could neither speak nor swallow; her breathing was much oppressed; the pulse low and contracted; her complexion saturnine; yet the abdomen was not much collapsed." P. 21.

Volatiles were applied to the olfactories, and antispasmodic frictions to the neck. In two hours she could swallow, and then an analeptic mixture was prescribed, with some musk every two hours. Towards evening, things wore a better aspect. The night was passed with varying symptoms, yet more tranquilly, and without fever or other accident. On the morning of the fifth day she was much better; and as there appeared a considerable suppuration, the dressings were taken off, but the ligatures were not removed. From this time the patient daily improved. The lochia were discharged as they ought; the milk appeared in the breasts. On the tenth day, some of the ligatures came away. In seven weeks after the operation the menses reappeared, though irregularly; but afterwards they came at their proper periods.

In the twelfth week, the patient paid her *deliverer* a visit, in the best health, bringing with her an admirably handsome and stout babe.

Dr. Locher is well entitled to a civic crown; but his feelings on the aforesaid visit must have afforded him more real pleasure than wreaths of laurel, or a triumphal arch!

---

N.] *Singular Case of Expulsion of a blighted Fœtus and Placenta at Seven Months, a living Child still remaining to the full Period of Utero-Gestation.* By JOHN CHAPMAN, Esq.

[ Med. Chir. Trans. Vol. IX. ]

THIS is a Case more curious than instructive, as the author modestly confesses; yet it is not undeserving of record in the Archives of Parturiological Science.

A woman, in the seventh month of pregnancy, was taken suddenly ill, with a considerable flooding; and by the time Mr. Chapman arrived, had passed a mass which, on examination, was discovered to be a perfectly healthy placenta, of a five or six month's size. To this was attached the membranes also, quite perfect, but of a dirty yellow colour, flattened, and closely embracing a small fœtus, not larger than they are generally seen between the third and fourth month. Neither pain nor hæmorrhage succeeded, and she went to the full period of utero-gestation, when a fine girl was produced, and both parent and child did well.

---

### O.] *Case of Ruptured Uterus.* By CHARLES FRIZEL, M. D.

[ Transactions of the King's and Queen's Association, Vol. II. ]

BRIDGET FAGAN was admitted into the Lying-in-Hospital on the 22d of April, 1814, about thirty years of age, of small stature, spare habit; had six children before, and generally severe times. With the present child she had been in strong labour for nearly three nights. At this time Mr. Grattan visited her, and found her in a very exhausted state, apparently without labour; the child's arm a long time expelled, and the patient altogether in such a dangerous predicament that she was sent to the hospital. Her pulse was now feeble; she was affected with hiccup and severe vomiting. The child's arm was quite protruded, black, and tumified; the shoulder lodged a little lower than the upper aperture of the pelvis. Directed a little wine and water to be given to the patient. With some difficulty Dr. Frizel got his hand passed into the uterus, which was found closely encompassing the body of the child. The left foot was laid hold of and brought down into the vagina, while the arm was returned. Every effort failed to bring the foot farther than the external parts, though aided by a garter-noose over the ancle. Some hæmorrhage supervening, an attempt was made to bring down the other foot, but without success; the crochet was then hooked in the acetabulum coxendicis, but it gradually tore its way towards the knee. Dr. F. on attempting again to pass his hand into the uterus to seize the other foot, was surprised to find a soft floating fold, which he discovered to be the intestines of the mother: With great difficulty the child and placenta were delivered, when the rupture of the uterus extended from the upper part of the symphisis pubis to nearly the middle of the opposite part of the sacrum. The intestines were carefully pushed up, with some difficulty. Withdrawing the hand slowly, the lacerated edges of the rupture were brought to overlap one another, and it was supposed they kept their position, particularly as the womb had, by this time, contracted a little. She was settled on her couch, on the right side; an anodyne draught was given her. In two hours she seemed tolerably free from pain, and disposed to sleep. She was given five grains of calomel, and about two hours afterwards, senna and salts. This producing no effect, an injection was ordered, and in the evening the bowels were opened. Next morning she was not so well; complained of great soreness and pains in the hypogastrium, increased by pressure. Fomentations; venesection; enema. Not to be prolix, she gradually recovered, and was discharged convalescent from the hospital on the 8th day of May, 1814. She has had another child, and enjoys good health.

Well may Dr. Frizel say, that it is impossible for any one (excepting those immediately engaged in the obstetric department of the profession) to form an opinion of the untoward and extraordinary difficulties, which sometimes occur in these trying scenes, where the best directed efforts and rules of art are too often frustrated; and where it only remains to act according to the best of our discretion in the discharge of our duty.

---

P.] *Case where Oil of Turpentine was used.* By WHITLOCK NICHOLL, M. D.

[ Transactions of the King's and Queen's College of Physicians, Vol. II. ]

ON the 27th of August, 1817, Dr. N. visited a girl, twelve years of age, whom he found with a hurried, feeble pulse; black tongue; distended bowels. She had been much purged, without medicine, and afterwards by mercurial and other laxatives. Mercurial pill and saline aperients ordered; but before these were administered she voided an immense quantity of black grumous fæces. By medicines of different kinds the secretions were brought to a tolerably healthy state, and the tongue became clean, yet still the following symptoms remained for a fortnight: Constant drowsiness; great disinclination to speak; tremulous debility; incessant irritation in the nose; dark red circumscribed hue on each cheek; pulse frequent; skin dry, and intensely hot; appetite ravenous; death-like coldness of the hands; emaciation. The least quantity of wine or other stimulant lighted up high febrile excitement. Various medicines of the purgative class were tried, but without effect. On the 17th of September Dr. N. exhibited ℥iß ol. terebinth. which dose was repeated in two hours; two hours after the second dose, a similar quantity was thrown up by glyster, while half an ounce of the oil was given by the mouth. These produced two copious stools, but without any thing worthy of notice. From this time, however, all her strange and anomalous symptoms disappeared as though by magic, and the patient rapidly recovered her former health.

Dr. Nicholl suspects, and we think justly, that the cause of this young lady's symptoms was seated in the alimentary canal. No appearance of tænia could be perceived. We every day, indeed, see a host of symptomatic and otherwise unaccountable symptoms resulting from disordered secretions in the bowels themselves, especially in what are denominated nervous or irritable females; and all will disappear occasionally, as in the above case, by properly adapted purgatives.

In the same volume Dr. Nicholl relates a case of angina pectoris, in an elderly gentleman who had been subject to constipation and flatulency for many years, and who had latterly experienced some mental anxiety in consequence of the loss of an only grand-child. He had difficulty in ascending any eminence, which caused him to stop, from a painful sensation across the chest, that left him on standing still. He had great flatulency and eructation. His seizures generally came on about eight o'clock in the evening, consisting of pain at and under the extremity of the sternum, not increased by pressure, attended with numbness and coldness of the arms, more especially the left arm, extending to the wrists. He knew his complaint was angina pectoris, and that he should die of the disease. He had no cough nor difficulty of breathing, or of lying on either side; no palpitation; no loss of appetite. Dr. Nicholl saw him in a paroxysm, and the pulse was unaffected. Whenever he was thoroughly purged by senna and manna draughts, he escaped the evening paroxysm, and at all times an opiate draught quickly relieved it. After taking more than usual horse and carriage exercise, on the 9th, 10th, and 11th of October, he had a very severe seizure with extreme mental agitation, and discharge of blood from the lungs. He was put to bed, had an opiate draught, and a blister to his chest. His night's rest was bad; several doses of senna and manna were given next day, and brought away very sour stools. He was harrassed constantly by strangury, which probably proceeded from the blister.

On the evening of the 14th, precisely at eight o'clock, the usual hour of attack, he said the fit was coming on, and became extremely agitated; complained of coldness and numbness of the hands; muttered some words indistinctly; and, on recovering a little, retired to bed, with the conviction that he had narrowly escaped a paralytic seizure. He took his opiate draught, and passed a tolerable night.

During the 15th he was unusually well, and in good spirits at the prospect of visiting Bath. When the dreaded hour of eight was struck, he got up from his chair, saying, "it was coming on." He walked up and down the room two or three times, in great agitation; then dropped on his knees, laid his head on a chair, called for his anodyne, and instantly expired. Leave was not obtained to inspect the body.

Such is the awful and sudden termination, in general, of this dreadful disease; two such terminations we have painfully witnessed in the course of the last four years. We shall recur again to the subject in a succeeding number of this Journal.

---

### Q.] *Cases of Inflammation and Enlargement of the Pancreas.* By Drs. Percival and Crampton.

[ Transactions of the Irish College, Vol. II. ]

Post mortem researches prove that organic diseases of the pancreas are comparatively rare, and it is evident that the difficulty of ascertaining the condition of this viscus, in the living body, by any other symptoms than such as are secondarily manifested through the medium of the stomach, liver, or some neighbouring part, must render our judgment, in many cases, uncertain, and our practice erroneous.

It is true, that when pain and tumour of the pancreas are mistaken for inflammation of certain parts of the liver, the measures of depletion, which are applicable to both, may dissipate the disease, before the error of diagnosis can be discovered. But the confounding the pain and jaundice caused by the pressure of an inflamed pancreas upon the ductus communis choledochus, with *spasm* or calculous obstruction of that canal, would be a more serious error; and it is with the view of preventing such mistakes and their consequences, that the distinguished physicians, abovementioned, bring forward the following cases.

*Case* 1. An elderly and rather corpulent gentleman had been frequently seized, in the course of last autumn, with acute pains in the epigastrium, and soreness and swelling of the parts, followed by jaundice. Cordials and mercurial purgatives were prescribed by his medical adviser, which relieved, but did not remove the urgent symptoms. Shortly after this he began to experience obscure pain in the chest, when making any bodily exertion, unattended by cough or respiratory disturbance. As this was considered symptomatic of the epigastric uneasiness, a blister was applied to the seat of the latter, with benefit. No blood-letting, local or general, had been resorted to.

Dr. Percival saw this gentleman on the 10th of November, at Bath, when his complexion was dingy and yellow about the eyes; tongue much furred; urine scanty and high coloured; pulse 78; epigastrium prominent, especially in the centre; the verge of the liver pretty clearly ascertainable, bearing free pressure, and exhibiting no sign of organic disease.

"But detached from this organ, in the gastric region, was an unusually dense mass, tender to the touch, and yielding slowly on pressure."

There was chilliness in the evening, and progressive emaciation. Eight leeches applied to the epigastrium bled copiously, and produced faintness. The tumour and tenderness were considerably reduced; the urine came free and natural; the pulse fuller and softer; and the patient greatly relieved. A vesication kept upon the same parts for ten days, with regulated diet, and saline aperients, considerably restored the patient. Still a tumour and hardness were perceptible in the epigastrium, until, a spontaneous diarrhœa occurring, it shrunk rapidly in its dimensions, "and before the expiration of a fortnight, entirely disappeared."

Dr. Percival's reasonings on this case, are ingenious and specious; but we confess we are somewhat sceptical respecting the pancreatic nature of this tumour. At the same time, we are free to acknowledge, that we are unable to offer a better explanation.

The following cases certainly tend to support the probability of Dr. Percival's opinion. The first was communicated to Dr. P. by Dr. Haygarth.

*Case* 2. A middle-aged gentleman had jaundice and bilious vomiting, with a disordered secretion of urine. The epigastrium was distended, and a hard tumour was distinctly felt, protruding from the middle of that region. Mercurial purgatives, and various cordial and diuretic medicines were employed. The patient became extremely dejected and feeble, even to deliquium. Blood, and at length fœtid pus were discharged by stool. Anasarca followed, and the patient died in three months.

The pancreas was found greatly enlarged, and occupying the place of the tumour felt in epigastrio. The ductus communis choledochus was imperforate in the parts adjacent to the pancreas, and where its pressure had been the greatest. The substance of the pancreas was scirrhous, and, on being cut into, was found to contain a considerable abscess. The liver was diseased.

*Case* 3. By Dr. Crampton. A lighterman, 35 years of age, addicted to drink spirituous liquors, complained, about five weeks before his admission, of incessant cardialgia, with flatulent eructations and constipation. Swelling in the epigastric region followed; and enlargement over the abdomen, with evident fluctuation, and anasarca of the legs. The skin became deeply tinged yellow; complained of severe obtuse pain, deep-seated in the scrobiculus cordis, shifting occasionally over the abdomen; respiration unaffected; pulse irregular and intermitting; no appetite, except thirst; stools clay-coloured; urine deep yellow brown, and scanty; skin dry; face emaciated. The patient died on the 12th of March, after a protracted and ineffectual treatment.

*Dissection.* The abdomen contained a large quantity of yellow serous fluid; liver was diseased, with small vomicæ, containing purulent matter. Pancreas hard and enlarged, particularly its head, which pressed on the gall ducts; gall-bladder full of ink-coloured bile; as also ductus communis choledochus; no bile in the intestines.

*Case* 4. Mr. C. aged 32, addicted to the ingurgitation of spirits, and intemperance in eating, had been using oxymur. hyd. for supposed or real syphilitic pains in the head, and in the cylindrical bones. After exposure to cold, subsequent to the warm bath, he was seized [22d July] with thirst, nausea, slight vomiting, and flatulent distention of the stomach.

26th July. Dr. Crampton found the abdomen swelled, with evident fluctuation; legs œdematous; circumscribed hard enlargement in the epigastrium, nearly circular, with a defined margin, tender on pressure, pulse natural; mouth sore from the mercury; diarrhœa with some blood; urine lateritious. The case appeared to Dr. Crampton to be that of enlarged pancreas, and only to be remedied by vigorous depletory treatment. Venesection to ℥xij. twelve leeches to the epigastrium. Blood highly inflamed. Jalap and supert. potass. at moderate intervals. Following day, considerable detumescence of the epigastrium; margin of the tumour more defined; urine less loaded.

On the 29th. The same depletions were repeated, and the blood exhibited the same appearances.

On the 2d of August, the dropsical swelling had disappeared, and the epigastric tumour, though still observable, was much diminished; urine clear and abundant. A blister to the epigastric region, and the opening powders to be continued.—By the 8th of August, all abdominal enlargement was gone, and his health much improved.

Whatever may be thought of the above cases, in respect to the exact nature of their pathology, they are very valuable land-marks to guide our course in a therapeutical point of view. A few years ago, abdominal dropsy would have been an effectual bar to the lancet; but now it is disregarded, whenever depletion is otherwise indicated.

---

R.] *On the Origin of Intestinal Worms, particularly the Ascaris Vermicularis.* By J. M. Barry, M. D. of Cork.

[ Transactions of the Royal Irish College, Vol. II. ]

Practical writers have been more intent on investigating the means of expulsion, than of the modes of production of intestinal worms, and thus inquiries have been checked, and Empiricism fostered on this interesting point.

It has never been satisfactorily proved that any species of intestinal worm, demanding the physician's attention, has derived its origin from an external source; yet, as the doctrine of *equivocal generation* is now abandoned by every *rational* Philosopher, the extraordinary metamorphoses which the lower classes of animals occasionally assume, and the instinctive motions which they exhibit for the propagation and preservation of their kinds, afford a strong presumption of some *ab externo* origin, which have hitherto escaped the natural Philosopher. The investigations of Drs. Baillie and Hooper have placed beyond a doubt, the distinction between *earth* worms and those found in the human bowels. The *Ascaris Vermicularis* is also still considered peculiar to man, since there is no well authenticated proof of its being found to live in any other medium. The proofs of the extrinsic origin of the *ascarides vermiculares,* Dr. Barry thinks, will be found in the following history.

In the year 1797, a gentleman and his family, residing near the town of Macromp, in Ireland, suffered severely from this species of worm; and upon examination, it was found that a spring, from which they constantly drank, was infested with worms which corresponded, in appearance, with the ascarides. They were not, however, anatomically compared; and therefore there is still a cloud of uncertainty hanging over the subject.

---

S.] *Account of a diseased Appearance in the Intestines of Children.* By JOHN CRAMPTON, M. D. &c.

[ Dublin Hospital Reports, Vol. II. ]

EVERY one conversant with Continental Medical Literature, knows that our brethren there have been much more attentive to the morbid appearances of the *mucous membranes* of the abdomen after fever, than we have been. They may have gone too far, and mistaken effects for causes; yet, their observations are highly entitled to our regard, especially the writings of M. Broussais. Dr. Cheyne has demonstrated, that the late epidemic fever in Ireland showed a strong tendency to attack the internal surface of the digestive organs; and an attention to this point must improve the practice in fever, where the epigastric distress is present.

" It shows, (says Dr. C.) that we should not confine our views merely to the *sensorium,* but extend them to other organs. Of the distress in these latter, the symptoms will often afford sufficient evidence; but the examination of those who die of such fevers will frequently put the matter beyond a doubt; indeed, the diseased appearances vary so much in those who die from fever, that it is natural to conclude, that no particular mode of treatment can be equally suited to fevers, as they occur attended by topical affections in different organs. The lancet and purgatives, so useful in certain forms of incipient fever, are by no means equally suited to such as are attended with gastric and intestinal distress, except in those instances where the *serous* membrane of the abdomen becomes inflamed, when it will be necessary to have recourse to general, as well as local sanguineous depletion." P. 288.

The disease, which is the subject of this paper, commenced with pyrexia, abdominal tenderness, loaded tongue, thirst, occasional vomiting; but more frequently diarrhœa, tenesmus, slimy and greenish stools, mixed with blood. If the disease was not too far advanced, the bowel symptoms gave way to appropriate treatment, on the subsidence of the fever. Where remedies proved ineffectual, a true dysentery became established—the tubercles on the mucous membrane were converted into ulcers, and the patient died in a hectic emaciated condition.

When the disease terminated favourably, a quantity of yellowish branny scales were passed in the stools, which floated on the surface of the fluid discharges. They were like minute portions of wax from honey-comb.

Several cases, in illustration, are recorded by Dr. Crampton. In the *first* of these, the mucous membrane of the large intestines was much diseased, being highly vascular, and covered with yellow granulations. Minute inspection, with a glass, shewed the villi converted into rounded tubercles, of a firm texture, and yellow colour; confluent in a few patches, but generally distinct. The *coats* of the intestines, when cut, were observed to be hard and thickened; but the submucous and cellular tissues were not engaged, and externally the intestines did not appear diseased.

This boy was admitted into hospital labouring under fever, combined with severe pectoral symptoms; his abdomen very tumid and tender to the touch; eyes weak and watery; a rash on the skin; pulse 128.

The *second* case related was a girl, ætat. 8, who passed through the fever in hospital, but fell a sacrifice to the small-pox during the convalescent stage.

*Dissection.* The villous coat of the stomach was found plaited, and, near the pylorus, studded with granules, probably the mucous glands. The large intestines were much diseased. The internal surface of the sigmoid flexure of the colon, when cut open, was covered with a black, fœtid, and gangrenous matter, which washed off with water. The prominent folds of the mucous membrane beneath, seemed studded closely with small dirty, white graulations, which extended up to the transverse arch of the colon, where the mucous membrane was extremely vascular.

The morbid appearances in the other cases, so much resemble the above, that we do not deem it necessary to state farther particulars here.

Dr. Crampton does not mean to infer that the disorganizations, in question, should, in every instance, be considered as the *cause* of the fever. It is probable, that in most instances, they existed antecedent to fever, especially in those who were exposed to contagion.

We hope our brethren, in these islands and its dependencies, will, in the inspection of bodies, not content themselves with an examination of the peritoneal surfaces of the abdominal viscera; but accurately ascertain also, the condition of the *mucous* membranes lining the interior of the intestinal track.

